# Perforated Bladder as a Cause of Abdominal Ascites in a Patient Presenting with Acute Onset Abdominal Pain

**DOI:** 10.7759/cureus.1241

**Published:** 2017-05-11

**Authors:** Raj Shah, Madhuri Ramakrishnan, Beenish Ahmed, Khalil Abuamr, Osama Yousef

**Affiliations:** 1 Department of Internal Medicine, University of Missouri Kansas City (UMKC); 2 Department of Internal Medicine, Baylor College of Medicine; 3 Gastroenterology, Truman Medical Center, University of Missouri School of Medicine, Kansas City, MO, USA

**Keywords:** ascites, neurogenic bladder, perforation, multiple sclerosis, acute kidney injury

## Abstract

Bladder perforation, especially when atraumatic, is a rare cause of ascites and is often difficult to differentiate from other causes of abdominal pain and ascites in the acute setting.

A 43-year-old Caucasian woman with a history of multiple sclerosis presented with acute abdominal pain. A computed tomography (CT) scan of her abdomen and pelvis without contrast revealed ascites, acute kidney injury (AKI) was noted on laboratory workup, and very little urine was drained by Foley catheter. Over the next several days, the patient's clinical condition deteriorated with no definitive diagnosis. A repeat CT of her abdomen and pelvis without contrast showed worsening ascites. She underwent paracentesis, which revealed a markedly elevated ascitic fluid creatinine consistent with bladder rupture. She then underwent an urgent cystogram to confirm the diagnosis, and the urologic consultant recommended conservative management with a Foley catheter to allow for bladder healing. Conservative treatment failed however, and she underwent a surgical repair with drain placement which was followed by an improvement in her clinical condition.

This case illustrates a unique presentation of a young woman with multiple sclerosis whose bladder perforation presented as abdominal pain and ascites. The multidisciplinary approach required here highlights the difficulty in reaching this diagnosis which is often undermined in patients who lack a history of traumatic injury. Such delays led to a complicated hospital course for our patient. Maintaining a broad differential for abdominal pain and ascites is essential.

## Introduction

Bladder rupture is usually considered in cases of abdominal trauma. Atraumatic bladder rupture is a very rare occurrence but is associated with a high morbidity and mortality, and the clinical course if often complicated by a delayed diagnosis [1]. For patients who present with abdominal ascites, the differential is broad, and the common causes explored first are liver disease, kidney failure, heart failure, and cancer. Atraumatic bladder rupture is usually not considered in the initial differential. Our case highlights the complexity of diagnosing and managing ascites secondary to bladder rupture.

## Case presentation

Our patient is a 43-year-old Caucasian woman who presented to the emergency department with severe, sharp lower abdominal pain that began immediately after voiding. She experienced associated symptoms of nausea, vomiting, and increased urinary frequency and urgency. She had no history of trauma to the abdomen, but she did have a history significant for untreated multiple sclerosis and a previous ovarian cyst rupture status-post right oophorectomy. In the emergency department, her physical exam revealed a soft, non-distended abdomen, with tenderness on palpation throughout the lower quadrants, but without guarding, rigidity, or rebound tenderness.

On initial presentation, she underwent a computed tomography (CT) scan of her abdomen and pelvis without contrast (she was allergic to contrast dye). The scan revealed ascites. According to the patient, she had been diagnosed with pelvic ascites of unclear etiology at an outside facility one year prior to presentation while experiencing similar symptoms. Pelvic ultrasound was only significant for a small left para-ovarian cyst (2.0 x 1.2 x 1.5 cm in dimension), and a partially visualized bladder appeared normal. Following gynecological consultation, there was a concern for a ruptured hemorrhagic cyst and conservative management was recommended until the episode resolved. Concurrent workups for pyelonephritis was negative. The patient developed an acute kidney injury (AKI) and urinary retention with a bladder scan revealing 584 mL of retained urine. However, straight catheterization drained only 250 mL.

**Figure 1:**
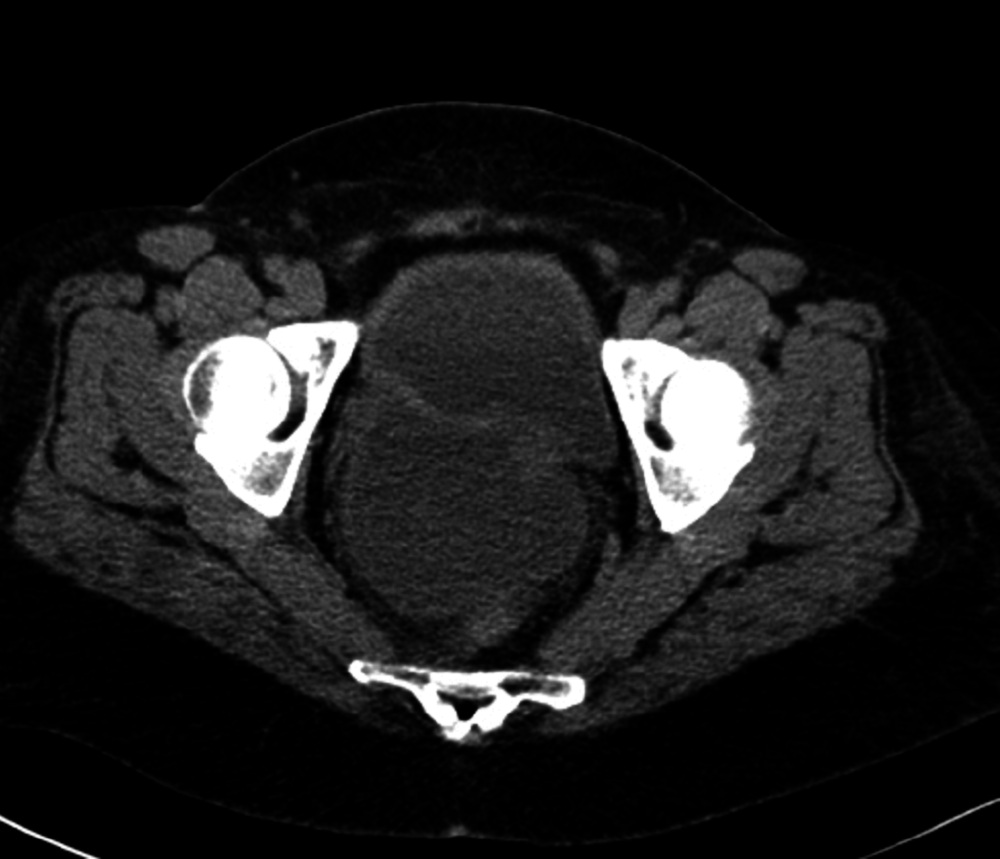
CT abdomen and pelvis without contrast showing bladder diverticulum, cannot rule out bladder rupture

Over the next few days, she began to complain of epigastric pain with worsening nausea and hematemesis, and had difficulty breathing. Her chest x-ray showed bilateral pleural effusions and atelectasis. The gastroenterologic consultant planned to perform an esophagogastroduodenoscopy, but the procedure was canceled due to a rapid deterioration of her condition, with worsening abdominal distension, sluggish bowel sounds, worsening AKI and a new anion gap metabolic acidosis. A second CT of her abdomen and pelvis without contrast showed a marked increase in abdominal ascites as well as a new abnormality of the bladder wall that likely represented a bladder diverticulum, but a bladder wall rupture or diverticular rupture could not be excluded (Figure 1). A fluid analysis was recommended based on the CT findings, and 4.5 L of straw-colored fluid was removed through paracentesis. There was a concern of secondary bacterial peritonitis from her low total protein count of 1.3 mg/dL, low glucose at 36 mg/dL, and of perforated or ruptured viscus from her elevated fluid creatinine at 8.27 mg/dL.

**Figure 2:**
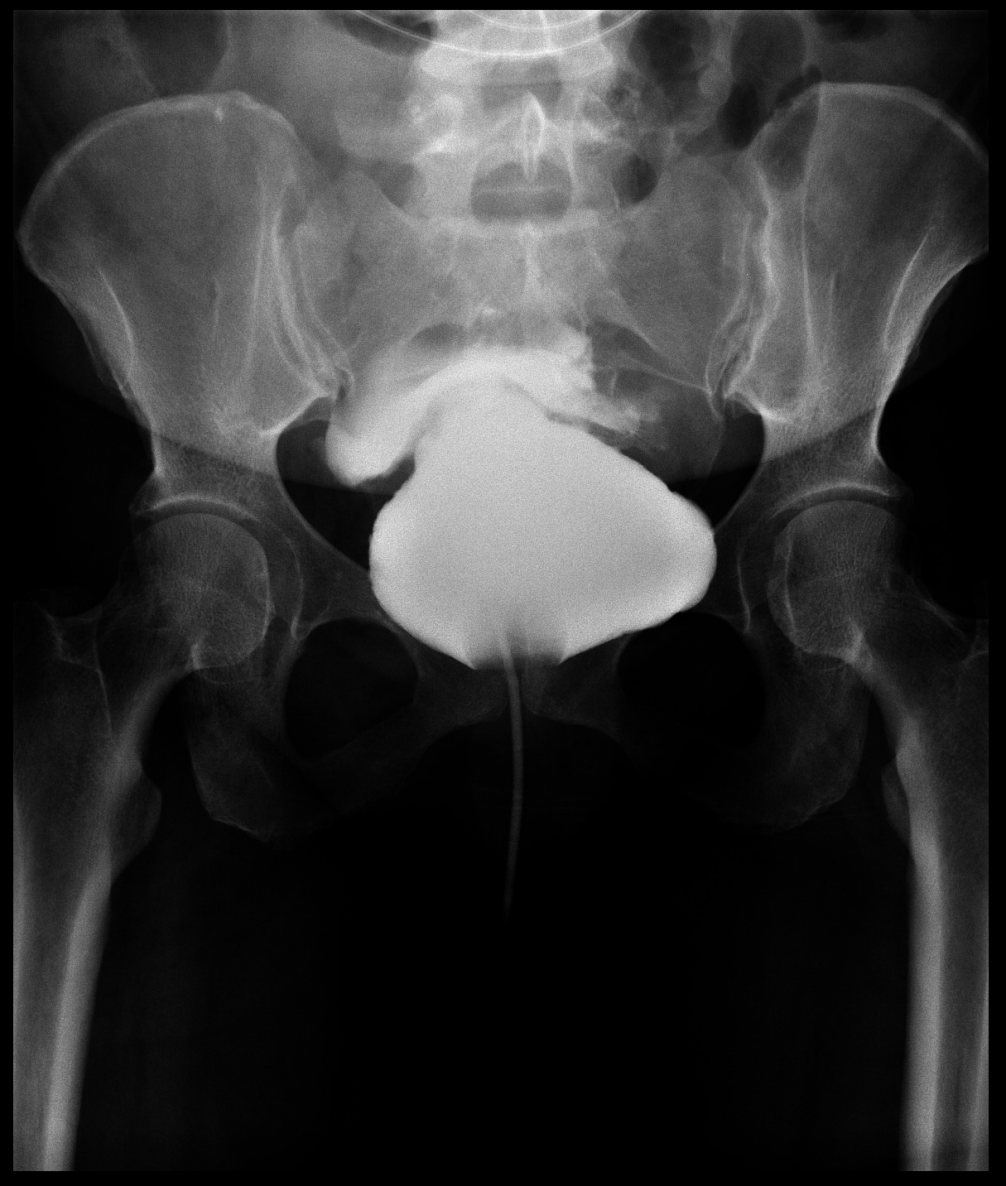
X-ray cystogram showing bladder rupture

Due to her declining urine output, low urine volumes were seen in her bladder scan with high ascitic fluid creatinine; a cystogram was immediately performed due to concerns of bladder rupture. The cystogram confirmed this diagnosis (Figure 2). Her serum creatinine prior to paracentesis was 3.5 mg/dL and dropped to 0.61 mg/dL the next morning. The cause of the patient’s bladder rupture was thought to be obstructive uropathy secondary to neurogenic bladder from her untreated multiple sclerosis.

The urologic consultant recommended nonsurgical, conservative management with postobstructive diuresis by a Foley catheter for 14 days to allow adequate healing of the bladder wall. However, the conservative management approach failed, and the patient experienced increased abdominal pain and ileus and, subsequently, underwent cystoscopy, cystorrhaphy, and diverticulectomy with drain placement.

The patient’s pain markedly improved postoperatively. The drain was removed, and she was discharged with a 2-week follow-up in the Urology clinic.

## Discussion

While traumatic rupture of the bladder is a more common occurrence, ascites secondary to a spontaneous bladder rupture is rare and documented sparsely in case reports. Due to the delay in diagnosis, patients have poorer outcomes and a high mortality rate. Cases have previously been reported secondary to a state of chronic inflammation, bladder outlet obstruction, bladder malignancy [1], as well as pregnancy.

Multiple sclerosis, through its disseminated, patchy demyelination in the brain and spinal cord is known to cause bladder dysfunction, including urinary retention, which is likely from decreased sensation of filling. A PubMed search found two other cases of bladder rupture in multiple sclerosis patients in a paper written in 1977 [2]. Both cases were of women who presented with peritonitis. In contrast, our patient did not present with features typical of acute abdomen, though her presenting complaint was abdominal pain.

With urinary ascites low on the list of most physicians’ differential diagnoses, it is not surprising that patients often go undiagnosed and face a more complicated hospitalization as a result. Failure to diagnose spontaneous bladder perforation within the first 24 hours contributed to a mortality rate of 25% [3]. The testing modality of choice is a CT cystogram and retrograde cystoscopy, while having a high creatinine level in the ascitic fluid increases the index of suspicion [4]. However, such tests are not routinely ordered unless there is a concern for urinary ascites.

The AKI or rise in serum creatinine in such cases of urinary ascites from bladder rupture is due to the process of “reverse auto dialysis” by the peritoneal membrane, which allows the diffusion of urea and creatinine from the ascitic fluid into the blood, causing the elevation of serum urea and creatinine [4].

Management can be conservative or invasive—both of which were demonstrated during the care of our patient. Our patient was fortunate her symptoms resolved despite the delay in reaching the diagnosis due to the complexity of her presentation. However, urinary ascites should be considered as a differential for someone presenting with abdominal ascites, especially when a more common cause is not obvious on presentation.

## Conclusions

Atraumatic or spontaneous perforation of the urinary bladder should be considered in the differential diagnoses in patients presenting with new onset ascites and AKI without any evidence of chronic liver or heart disease or other common causes of ascites. Early diagnosis can greatly improve the patient’s hospital course and overall outcome.
